# Temporal and spatial variability of dynamic microstate brain network in disorders of consciousness

**DOI:** 10.1111/cns.14641

**Published:** 2024-02-22

**Authors:** Yaqian Li, Junfeng Gao, Ying Yang, Yvtong Zhuang, Qianruo Kang, Xiang Li, Min Tian, Haoan Lv, Jianghong He

**Affiliations:** ^1^ Key Laboratory of Cognitive Science of State Ethnic Affairs Commission, College of Biomedical Engineering South‐Central Minzu University Wuhan China; ^2^ College of Foreign Languages Wuhan University of Technology Wuhan China; ^3^ Department of Neurosurgery, Beijing Tiantan Hospital Capital Medical University Beijing China

**Keywords:** disorders of consciousness, electroencephalography, microstates, mutual information

## Abstract

**Background:**

Accurately diagnosing patients with the vegetative state (VS) and the minimally conscious state (MCS) reached a misdiagnosis of approximately 40%.

**Methods:**

A method combined microstate and dynamic functional connectivity (dFC) to study the spatiotemporal variability of the brain in disorders of consciousness (DOC) patients was proposed. Resting‐state EEG data were obtained from 16 patients with MCS and 16 patients with VS. Mutual information (MI) was used to assess the EEG connectivity in each microstate. MI‐based features with statistical differences were selected as the total feature subset (TFS), then the TFS was utilized to feature selection and fed into the classifier, obtaining the optimal feature subsets (OFS) in each microstate. Subsequently, an OFS‐based MI functional connectivity network (MIFCN) was constructed in the cortex.

**Results:**

The group‐average MI connectivity matrix focused on all channels revealed that all five microstates exhibited stronger information interaction in the MCS when comparing with the VS. While OFS‐based MIFCN, which only focused on a few channels, revealed greater MI flow in VS patients than in MCS patients under microstates A, B, C, and E, except for microstate D. Additionally, the average classification accuracy of OFS in the five microstates was 96.2%.

**Conclusion:**

Constructing features based on microstates to distinguish between two categories of DOC patients had effectiveness.

## Graphical abstract

This is the first study to explore the temporal and spatial variability of dynamic microstate brain networks in disorders of consciousness. Our findings suggest that constructing features based on microstates to distinguish between two categories of disorders of consciousness (DOC) patients has effectiveness. The stronger activation of specific brain regions in different microstates in vegetative state patients suggests that their level of consciousness may have been underestimated. Our findings may contribute to the neurophysiological mechanisms underlying human consciousness as well as provide new insights to reveal the level of consciousness in DOC.

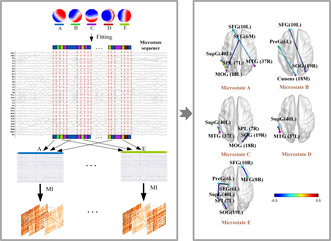

## 1 INTRODUCTION

Consciousness depends on normal large‐scale network function in the brain.^1^, ^2^ Patients who survive brain injuries may develop disorders of consciousness (DOC). DOC is clinically divided into the minimally conscious state (MCS) and vegetative state (VS). Patients in the MCS respond poorly to stimuli, but show intermittent conscious behavior, while the VS results from diffuse lesions of the thalamus, cortical neurons, or the white matter tracts connecting them.^3^ Determining consciousness in noncommunicative patients through clinical examination is a crucial yet difficult task. The Coma Recovery Scale‐Revised (CRS‐R) score is commonly used in diagnosing DOC, but it has its limitations.^4^ Consciousness levels can fluctuate in the short‐ and long‐term, leading up to 40% of noncommunicative DOC patients being misclassified as being in a VS.^5^

Over the past two decades, various techniques such as electroencephalography (EEG) and functional magnetic resonance imaging (fMRI) had been adopted to aid in the clinical evaluation of patients with MCS and VS. Compared with fMRI, EEG is often used as a clinical auxiliary method due to its advantages of high efficiency and portability.^6^ EEG can record fluctuations on time scales and is more suitable for studying the temporal dynamics of the resting state and exploring its influence on stimulus processing. The increasing application of multi‐channel EEG to study the temporal and spatial properties of resting‐state networks is based on the concept of EEG microstates.^7^, ^8^ Microstates refer to the global field power of the EEG, which typically remains stable for 80–120 ms and can be described by a finite number of scalp potential topographies.^9^ Microstates highlight the notion that the scalp potential field reflects the transient state of global neuronal activity.^10^

The traditional approach to studying changes in brain networks in DOC patients is based on examinations of functional connectivity (FC) and spectral energy, and these changes have been shown to vary with levels of consciousness. It has been shown that patients with long‐term DOC have higher weighted signed mutual information (MI) connectivity between left and right parietal regions, and higher frontoparietal weighted phase lag index connectivity was found in the alpha band.^11^ Zheng et al.^12^ highlighted the role of thalamo‐cortical connectivity in patients' behavioral characteristics and levels of consciousness and suggested that diffusion tensor imaging combined with machine learning algorithms may facilitate diagnostic differentiation in DOC. However, the spatiotemporal dynamics and spatiotemporal interaction effects in DOC patients remain unclear. Recently, some researchers proposed an EEG microstate method to investigate the rapidly changing global state of the brain in DOC patients. Zhang et al. identified seven microstates with distinct spatial distributions of cortical activation and revealed significant differences in microstate properties between the MCS and VS groups, including spatial activation patterns, temporal dynamics, state transitions, and connection construction. Combined with microstate, entropy, power of α and δ frequency bands, and connectivity indicators,^13^ Stefan et al.^14^ selected the best feature subset to develop an automated system for severe DOC outcome prediction. The results showed that the constructed feature subset could provide high predictive power. Thus, brain connectivity and microstates provide complementary perspectives on the neural dynamics underlying DOC. At present, some studies have explored the neurodynamic characteristics of stroke patients, cyber sickness, and Alzheimer's disease by combining microstates and FC.^15^, ^16^, ^17^ However, there are few studies on the neural dynamics of DOC functional connectivity combined with microstates.

The brain functional connection of VS patients was less strong than that of MCS patients, according to several earlier studies.^18^, ^19^ The salience network and default network link of the DOC were also noted in some studies which have been somewhat compromised, whereas MCS had greater preservation than VS.^20^, ^21^, ^22^ Although numerous studies had demonstrated that VS patients had weaker FC under specific brain regions than MCS, the level of consciousness of patients classified as VS was significantly underestimated due to the high rate of misdiagnosis (40%) based on the differentiation between the two categories using CRS‐R. Currently, differential diagnosis of MCS and VS is an important challenge for physicians and neuroscientists. Extracting FC features based on microstates and constructing a classification system can throw light on distinguishing two types of DOC patients. Exploring the strength of connectivity information in the cerebral cortex between VS and MCS patients in each microstate deserves further study.

This study combined microstate and dynamic FC to examine the spatiotemporal state of the brain in DOC patients. In order to explore the neural mechanism behind each microstate, we mapped the EEG to each microstate and thus explored the FC differences between MCS and VS patients. Then two data analysis methods based on all channels and a few channels respectively were proposed. Analyzing brain network information differences based on fewer channels is the novel approach described in this paper. The optimal feature subset (OFS) obtained by feature selection and machine learning can assist in clinical differentiation between MCS and VS patients. The constructed MI functional connectivity network (MIFCN) provides new insights to reveal the level of consciousness in two types of DOC patients.

## 2 MATERIALS AND METHODS

### 2.1 Patients and diagnosis

This study acquired EEG from 32 DOC patients diagnosed with CRS‐R‐based VS or MCS from the Department of Neurosurgery, Beijing Tiantan Hospital, Capital Medical University. With an average age of 50.81 ± 11.88 years, the MCS group (*n* = 16) consisted of nine males and seven females, whereas the VS group (*n* = 16) had 10 males and six females (average age: 40.50 ± 14.69 years). The MCS group had a median age of 56 years (IQR 40–59), while the VS group had a median age of 40 years (IQR 30–52). The CRS‐R would be re‐administered five times in the week after the EEG recording to verify the consistency of clinical diagnosis.^4^ All participants provided informed consent prior to the start of the trial, and they were free to leave at any moment. The Declaration of Helsinki and all pertinent rules and regulations were followed during every procedure. This study was approved by the local ethics committee of Beijing Tiantan Hospital, Capital Medical University.

### 2.2 EEG recording and processing

Resting‐state EEG signals were recorded using a 10–20 system with a standard 32‐channel EEG recorder (Nicolet EEG V32, Natus Neurology, USA) equipped with sintered Ag/AgCl needle electrodes. The sampling frequency was 500 Hz and the impedance between the electrodes and the patient's skin was always kept below 5 kΩ to ensure a proper signal‐to‐noise ratio (SNR).

Data preprocessing was performed in MATLAB (version R2016a, MathWorks Inc., Natick, MA, USA) using the eeglab toolbox (version 2021.1). The preprocessing steps were as follows: (1) positioned the electrode spatially; (2) removed obvious noise segments manually, and used the eeglab toolbox to interpolate channels (spherical method) for replacing bad channels; (3) used notch filters to remove 50 Hz power frequency signals, and performed 1–40 Hz bandpass filtering; (4) downsampled the sampling frequency to 250 Hz to reduce computational complexity; (5) used independent component analysis (ICA) to remove oculoelectricity and myoelectricity; (6) regarded signals exceeding ±150 μV as ring segments and eliminated them by the absolute threshold method; and (7) took the average reference for re‐referencing.

### 2.3 Microstate clustering process

This prospective and observational study used Cartool software (https://sites.google.com/site/cartoolcommunity/) to perform microstate calculations on resting‐state EEG data of 32 DOC patients (extracted for 5 min per patient) after preprocessing. The data were filtered again at 2–20 Hz before calculation to remove the effect of high‐frequency noise. The highest spatial correlation was observed to exist between four and six types of microstates during numerous studies,^10^, ^23^, ^24^, ^25^ hence this was chosen as the clustering range. The specific process of microstate clustering could be divided into the following three steps.

As the first step, the *k*‐means algorithm was used to cluster microstates at the individual level. The global field power (GFP) of the raw data was first calculated, and GFP was equivalent to the spatial standard deviation of the average‐referenced signal across all electrodes. Since EEG topographies tend to remain stable during periods of high GFP, each peak of the GFP curve provided optimal topographic SNRs.^26^ Therefore, topographies at GFP peaks were representative of topographies at surrounding time points. The *k*‐means algorithm was performed separately on the topographies at GFP peaks. Combined with the meta‐criteria described in Custo et al.,^27^ the optimal number of microstate classes was determined for each participant, and a set of “individual topographic maps” was formed.

GFP defined the standard deviation of potentials across all electrodes at each sampling time point, defined as:

(1) $$\mathrm{GFP}\left(t\right)=\sqrt{\frac{\sum_{i=1}^{n}{u_{i}}^{2}}{n},}$$

where i represented each electrode, n was the number of electrodes (here n is 32), and u represented the measured voltage of each channel.

In the second step, the *k*‐means algorithm was used for clustering at the group level. Firstly, randomly selected a certain range of topographies from the individual topographies as the template topographies, and then calculated the pairwise spatial correlation between the template topographies and the individual topographies. Secondly, used the global explained variance (GEV) to perform quantitative evaluation until the template topography and individual topography had the greatest spatial correlation and the GEV reached stabilization. GEV was defined as:

(2) $$\mathrm{GEV}=\frac{\sum_{j=1}^{J}{\left[\mathrm{GFP}\left(j\right)C_{j,n}\right]}^{2}}{\sum_{j=1}^{J}{\left[\mathrm{GFP}\left(j\right)\right]}^{2}},$$

where $\mathrm{GFP}\left(j\right)$ was the global field voltage of individual topography j, $C_{j,n}$ was the spatial correlation between individual topography j and template topography n, and J was the number of individual topography.

**FIGURE 1:**
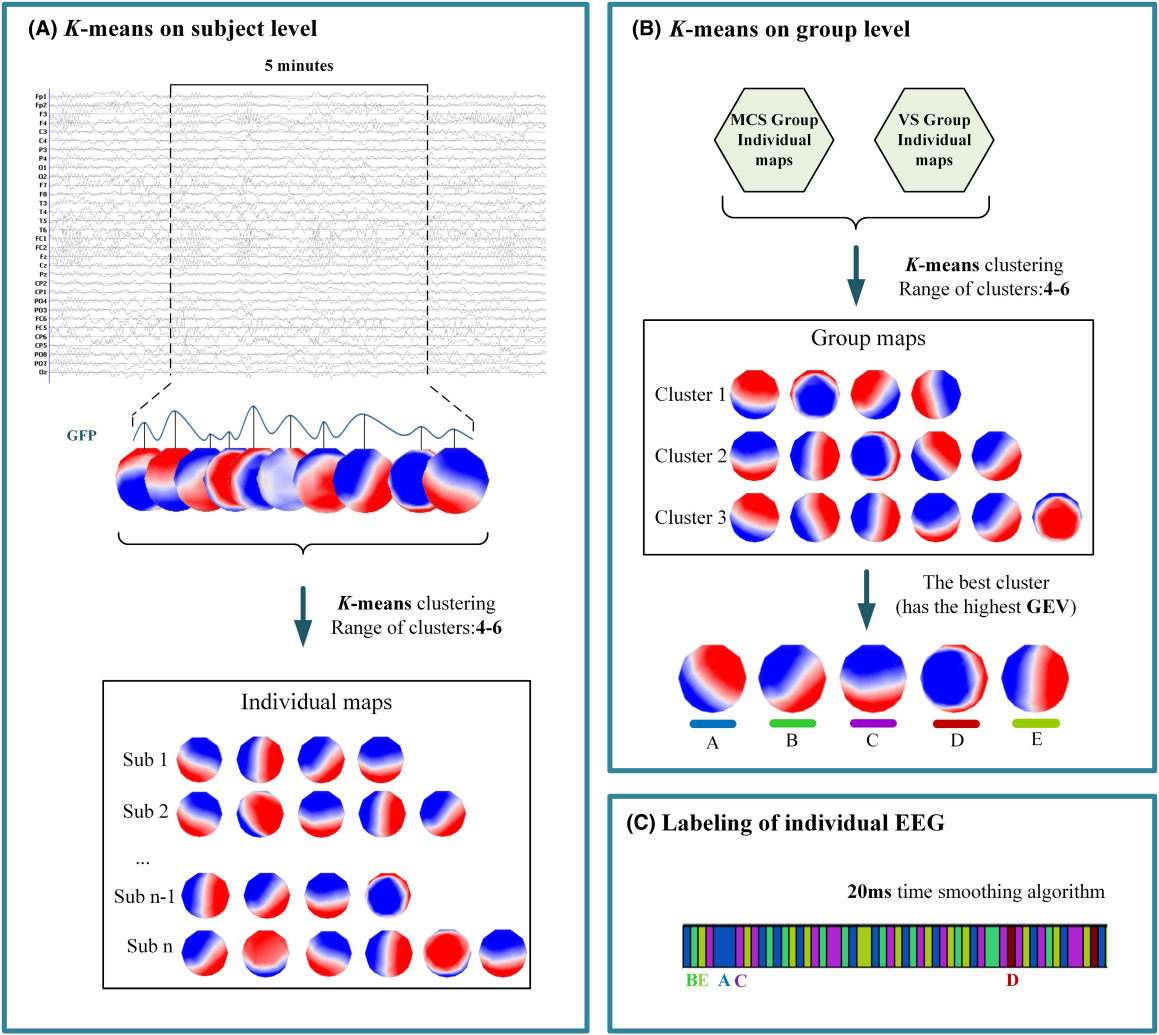
Microstate clustering flowchart. (a) For each subject, using the *k*‐means algorithm to obtain individual microstate maps. (b) The individual maps are combined to obtain group maps. (c) The sequence of microstate transitions for a single subject after fitting. Sub = subject.

In the third step, the template topographic map was fitted with the original EEG data with the obtained template topographic maps through the second step, by which each patient would get a microstate transition sequence. In addition, a time smoothing algorithm of 20 ms was selected in the fitting process, which could eliminate microstates with shorter time and assign them to adjacent microstates with higher spatial correlation. Figure 1 was a flowchart of microstate analysis.

### 2.4 Microstate‐based functional connectivity of brain networks

**FIGURE 2:**
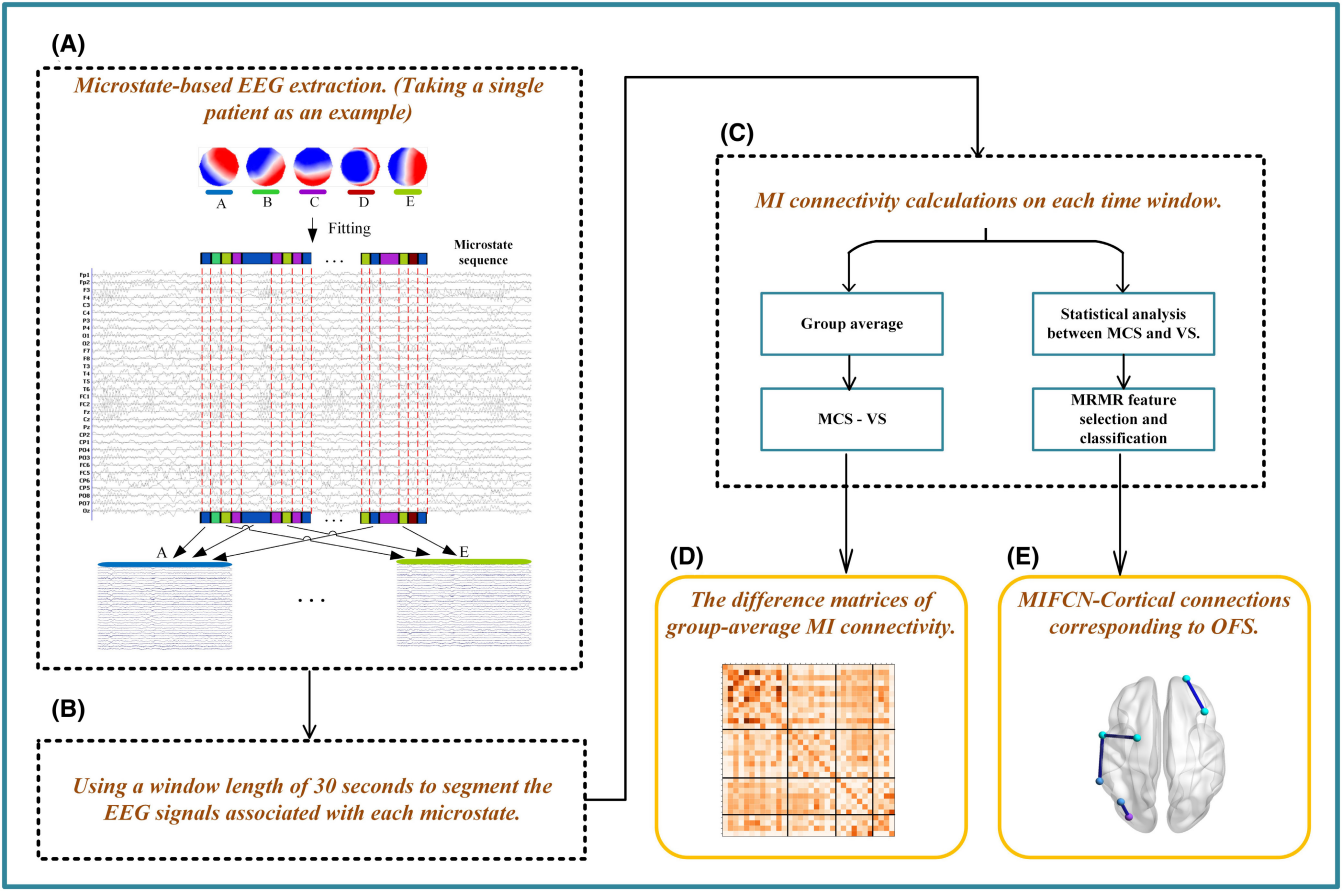
Flowchart of the proposed framework. The pipeline contains five major steps. The main methods are represented by the black dashed boxes, while the main results are indicated by yellow solid frames. (a) Microstate‐based electroencephalography (EEG) extraction method illustrated for a single patient. A through E represent the five microstates. (b) Using a 30‐second window length to segment the EEG signals corresponding to each microstate. (c) Mutual information (MI) connectivity calculations on each time window. One method focused on all channels averaged the MI connectivity matrices, and then performed a subtraction (MCS–VS). The other method, which focused on only a few channels, was based on feature selection and machine learning. (d) The difference matrices of group‐average MI connectivity. (e) The MIFCN‐Cortical connections corresponding to optimal feature subset.

Since the sampling frequency was 250 Hz, the 5 min of EEG contained 75,000 sampling points, and the microstate calculation gave each sampling point a state. Given the time smoothing algorithm made the duration of each state longer than 20 ms, each microstate in the state transition sequence contained at least five sampling points. Through Figure 2a, we can see that the EEG signal of each patient is segmented based on the microstate transition sequence, assuming that the microstate clustering template has five microstates. Since the duration of each microstate is different, the total length of time to extract the corresponding EEG based on the microstates is also different, which is shown in Table 1.

**TABLE 1:** The total time length (second) and time window count in each microstate.

Patients	TTL (TWC)				
	A	B	C	D	E
MCS	857.53 (28)	1184.66 (39)	1372.45 (45)	401.95 (13)	983.41 (32)
VS	1119.45 (37)	1018.68 (33)	1169.65 (38)	315.02 (10)	1117.20 (39)

Abbreviations: TTL, total time length; TWC, time window count.

In the course of our experimental investigations, it was observed that certain microstates within the transition sequence exhibited notably short durations. For instance, the EEG corresponding to some microstate A lasted only 1 s or even less. To enhance the SNR of the signal employed in calculating FC, dynamic functional connectivity (dFC) was utilized to segment the EEG corresponding to each microstate using a windowing approach (see Figure 2b). In contrast to resting FC, dFC allowed the study of functional connectivity changes at a finer scale, and it used sliding time window lengths to focus on the averaged results over all windows, which captured what characterizes spontaneously repeating FC patterns. However, the dFC window length should not be too large to capture worthless dFC changes, nor should it be too small to introduce unrealistic dynamics. This study used a window length of 30 s to divide the EEG signals corresponding to each microstate.^28^ The number of windows corresponding to each microstate is shown in Table 1. To investigate the dFC between the 32 channels, FC calculations were conducted on the EEG data at each time window within each microstate. The MI was utilized to measure the dFC and two data analysis methods were employed (see Figure 2c).

In information theory, MI was used to measure the interdependence between two random variables. The larger the value, the more correlated the two signals are, and vice versa. Given two random variables x and y, their MI was defined in terms of their probability density functions $p\left(x\right)$, $p\left(y\right)$ and $p\left(x,y\right)$:

(3) $$I\left(x;y\right)=\int \int p\left(x,y\right)\log\frac{p\left(x,y\right)}{p\left(x\right)p\left(y\right)}dxdy,$$

x and y represented the EEG signals of any two channels when applied to EEG analysis, and MI could measure the amount of information obtained from one channel to another.

### 2.5 Statistical‐based feature selection

**FIGURE 3:**
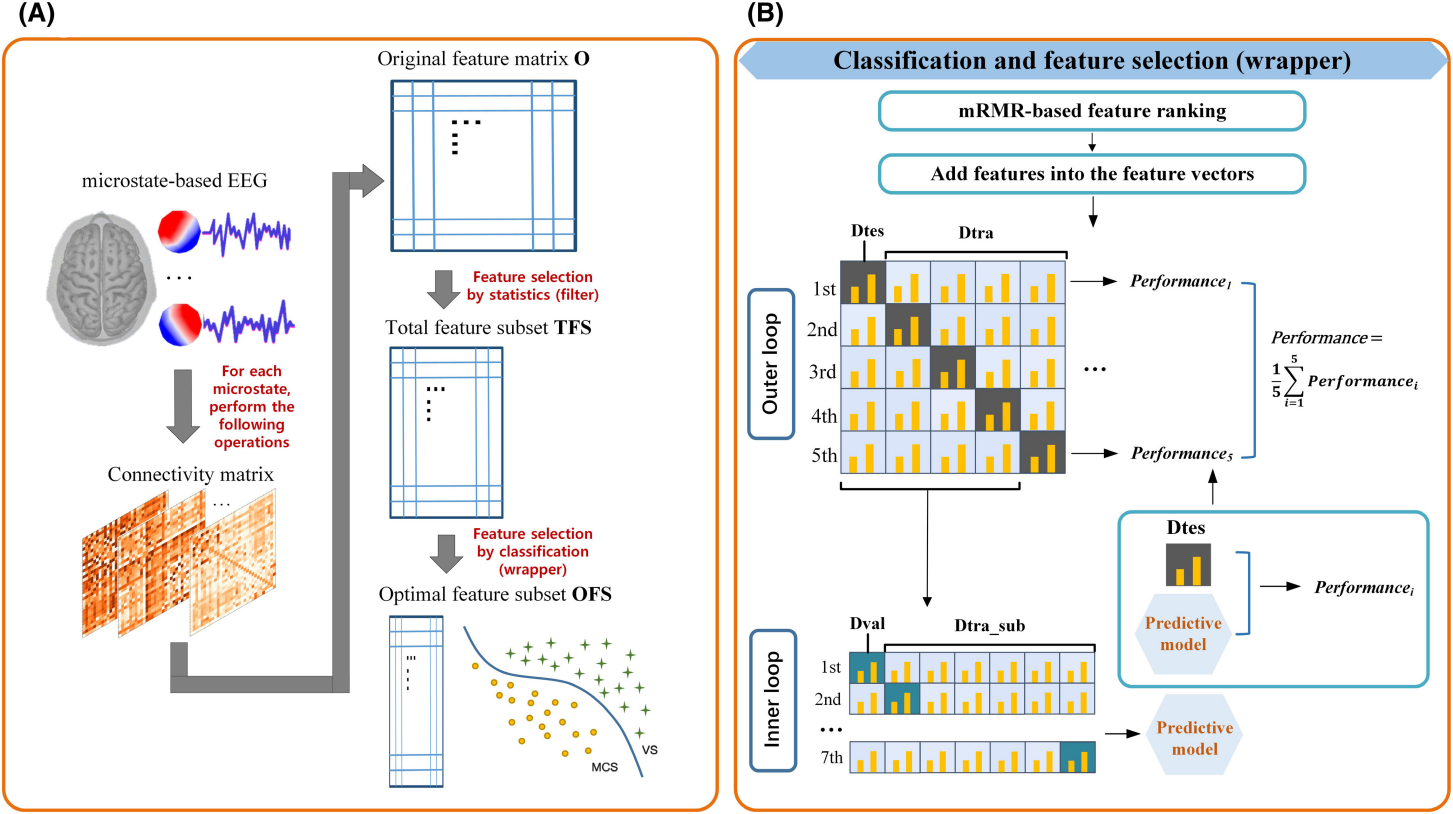
(A) Feature selection process. (B) Classification model with the wrapper feature selection.

In this study, feature selection to remove redundant features had two steps. The first step was conducted by statistics. We used The Shapiro–Wilk normality test to assess the data distribution, and all data were tested for normality. For the MI characteristics that met the normal distribution assumption, independent sample *t*‐tests were performed. For those that did not meet the normal distribution assumption, nonparametric independent sample Wilcoxon rank sum (Mann–Whitney) tests were conducted. Statistical significance was set at p<0.05, and all p values were corrected by a Bonferroni correction (p value <0.01/496) to control for false positives because multiple comparisons were used in each microstate. Since the MI connectivity matrix was symmetric, we took the upper triangular 496 elements for statistics to obtain the original feature matrix O. As a result, O had 496 columns, and the rows represented the number of samples, that is, the number of windows. Statistical analysis was then conducted on O, removing features that did not show significant differences. This resulted in a new total feature subset (TFS) (see Figure 3A). This procedure was considered as filter‐based feature selection. Subsequently, the classification‐based feature selection was performed.

### 2.6 Classification‐based feature selection

After applying the first step of filter‐based feature selection, an OFS was created using a wrapper strategy (see Figure 3B) for feature selection.^29^, ^30^ First, TFS in each microstate was ranked based on mRMR, and the top‐ranked new features were gradually added to the feature space to form a new feature vector. Then, a five‐fold cross‐validation (CV) strategy was applied to the ranked TFS, with the samples randomly divided into a training dataset (Dtra) and a testing dataset (Dtes) in a 4:1 ratio. The five iterations of the outer loop ensured that different samples served as independent test sets to improve the generalization ability of the model. Next, an internal seven‐fold CV was performed on the Dtra to overcome the overfitting problem, whereby Dtra was randomly divided into a training subset (Dtra_sub) and a validation dataset (Dval) in a ratio of 6:1. The inner loop was used to evaluate the model's performance metrics and hyperparameter values to ensure optimal performance on the training dataset. Finally, 5 predictable models were obtained, and independent Dtes were applied to the predictable models. The average of the five test results was then used to evaluate the classification performance. For the selection of the classifier, this study used the CatBoost model. CatBoost can address the gradient and prediction bias problems, and the algorithm has the advantages of resilience (cutting the need for significant hyperparameter tuning) and great performance. We utilized the CatBoost classifier with the following hyperparameters: 500 iterations, “MultiClass” loss function, tree depth of 6, and a learning rate of 0.1. These parameters were chosen to optimize the model's performance in handling the multi‐class classification task while controlling for overfitting and ensuring efficient convergence during training. In addition, the present study used the mRMR method to find the set of features in the original feature set that had the greatest relevance to the final output, but the least relevance between the features.^31^, ^32^

mRMR was a feature selection algorithm based on MI. By MI formula (3), feature relevance (*Rl*) and redundancy (*Rd*) were defined as:

(4) $$\mathrm{Rl}\left(S,c\right)=\frac{1}{|S|}\sum_{f_{i}\in S}I\left(f_{i};c\right),$$

(5) $$\mathrm{Rd}\left(S\right)=\frac{1}{{\left|S\right|}^{2}}\sum_{f_{i},f_{j}\in S}I\left(f_{i},f_{j}\right),$$

where S was the feature set, c was the sample class, and f was a single feature in S.

To maximize the correlation between features and minimize the redundancy, mRMR was realized through the Φ operation:

(6) $$\max\Phi \left(\mathrm{Rl},\mathrm{Rd}\right)=\mathrm{Rl}-\mathrm{Rd}.$$

Algorithm 1 Described the process of mRMR.

**Table:** 

**Algorithm 1** mRMR
**Require**: The feature set *F*, a number *m* of features.
**Ensure**: A feature subset $\left\{f_{1},\ldots ,f_{m}\right\}\subset F$
1: S=∅
2: **for** k = 1 to m **do**
3: $f_{k}\in \left\{\max\Phi |f\in F\right\}$
4: add $f_{k}$ to S
5: **end for**
6: **return** *S*

mRMR takes the forward search approach, and hence the variable S that holds the features selected at each iteration of the for loop (line 2–5) is initialized to the empty set (line 1). Then, for each iteration of the for loop, a single feature f that maximizes maxΦ is added to S. When all features were ranked by mRMR and validated by CV, the feature group (x≤m) of the top x features had the highest correlation and the lowest redundancy, which was named the OFS. This study used the average value of accuracy (ACC), true‐positive rate (TPR), and true‐negative rate (TNR) in each fold to measure the classification performance. Among them, ACC could evaluate the general predictive ability. TPR and TNR helped to evaluate the robustness to class imbalance. Define *TP*, *TN*, *FP*, and *FN* as correctly predicted samples (true positives and true negatives) and misclassified samples (false positives and false negatives) respectively, and the three evaluation indicators were defined as follows. Figure 3 presented the feature selection process and the classification model used in this study.

(7) $$\mathrm{ACC}=\frac{\mathrm{TP}+\mathrm{TN}}{\mathrm{TP}+\mathrm{TN}+\mathrm{FP}+\mathrm{FN}};$$

(8) $$\mathrm{TPR}=\frac{\mathrm{TP}}{\mathrm{TP}+\mathrm{FN}};$$

(9) $$\mathrm{TNR}=\frac{\mathrm{TN}}{\mathrm{TN}+\mathrm{FP}}.$$

## 3 RESULTS

### 3.1 Microstate clustering results

**FIGURE 4:**
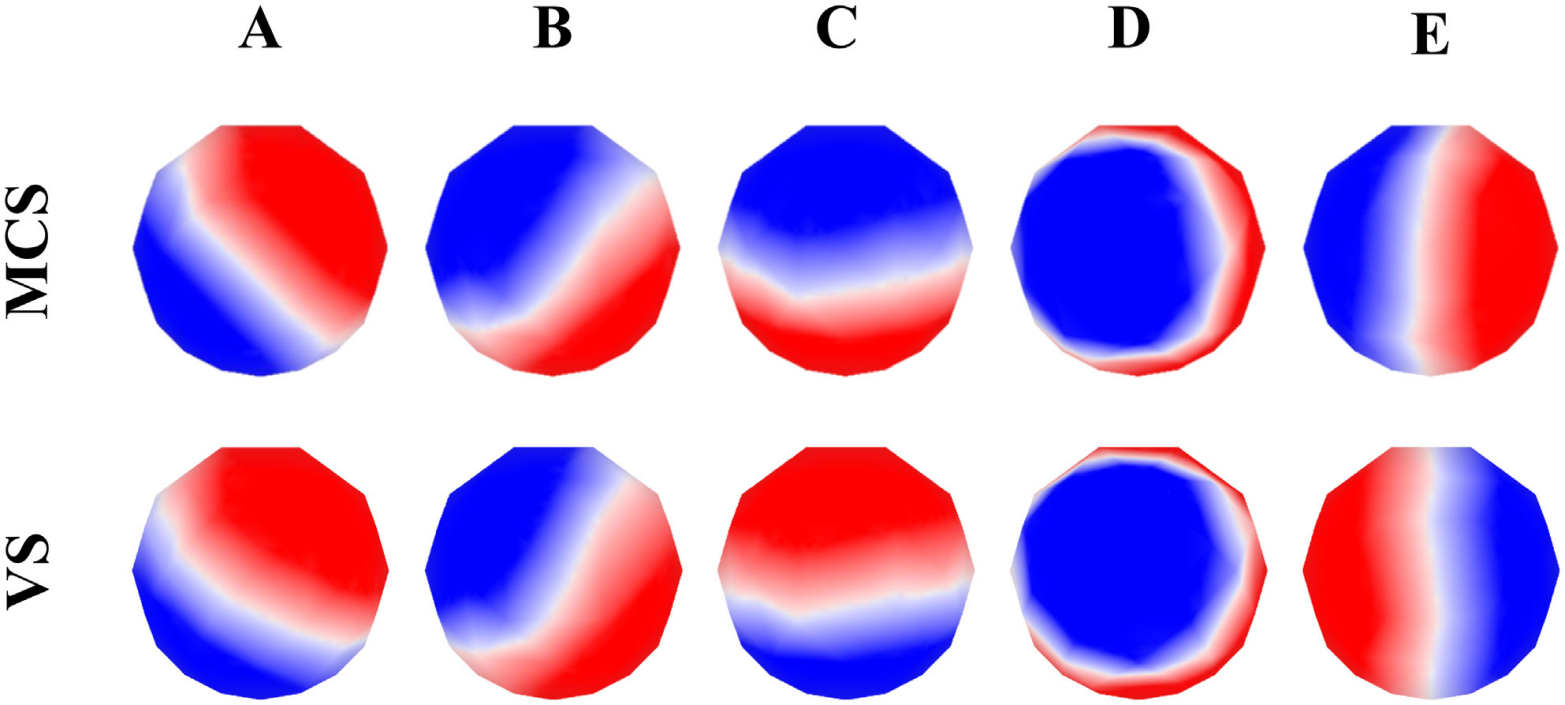
Microstate clustering templates for minimally conscious state group and vegetative state group.

After the microstate clustering analysis on the EEG data of two DOC groups, the microstate topological structure was obtained and shown in Figure 4. The GEV of these two groups of topological maps reached 72.60% and 73.45%. Since the *k*‐means algorithm ignores polarity, similar patterns but opposite colors can be regarded as the same microstate. For example, the microstate C and microstate E of two groups of patients can be regarded as the same microstate. It can also be seen from Figure 4 that microstate A extends diagonally from the left frontal lobe to the right posterior lobe, while microstate B extends from the right frontal lobe to the left posterior lobe and extends diagonally in the opposite direction to A. The line between the two polarities is horizontal in microstate C, and microstate D is usually a circular arc with the center point in the central region. These four microstates are highly consistent with the template topography in a large number of previous microstate studies.^10^, ^23^ However, in addition to these four classical microstates, this study has identified a new microstate that always appears steadily during clustering. It exhibits left–right symmetry and has been retrospectively classified as being associated with the cortex involved in the default network.^27^, ^33^ The default network is a crucial network in the field of DOC,^22^ and this microstate has been named as microstate E.

### 3.2 Mutual information functional connectivity matrix

In this work, we calculated the MI functional connection of the EEG in each time window for the five microstates. Thus, an array of H×H×N (where H = 32 represents the channel, N is the number of windows) was formed by connecting the estimated dFC values of each window within every microstate.

**FIGURE 5:**
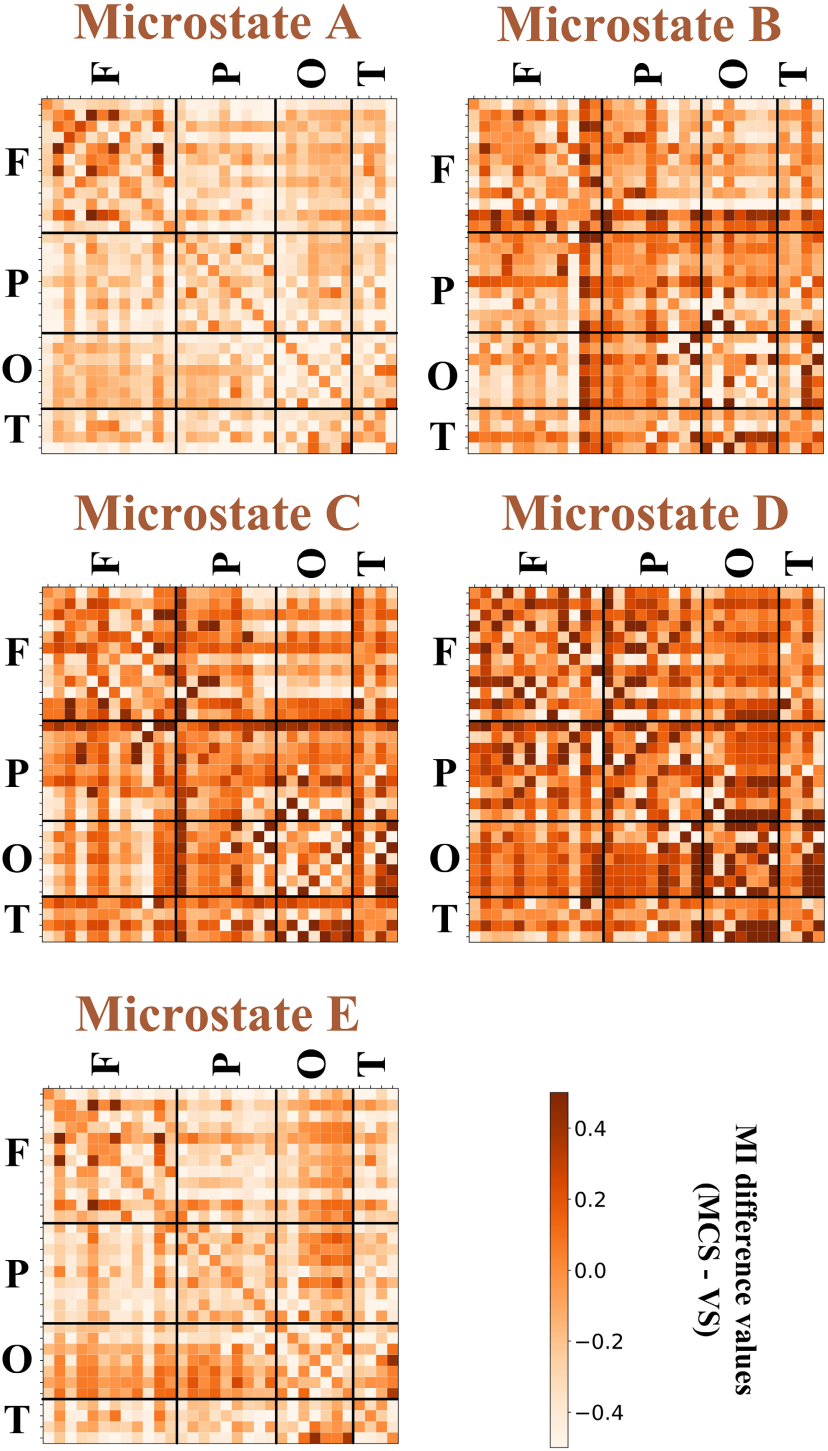
The difference matrices of group‐average mutual information connectivity for five microstates. Each row and column of the matrix represents 32 channels. The weight of the link from the horizontal electrode channel to the vertical electrode channel is measured by each node element and is color‐coded. The black solid line in the matrix divides the brain region into frontal lobe (F), parietal lobe (P), occipital lobe (O), and temporal lobe (T) according to the electrode channel.

This study averaged the MI connectivity matrix in each time window according to N, and then obtained the 32×32 group‐average connectivity matrices of the two patient groups for each microstate. Finally, the difference processing (MCS−VS) was performed, resulting in the difference matrices of group‐average MI connectivity (see Figure 5). Most of the values in the figure fall within the interval of [−0.5, 0.5]. Within the interval of [0, 0.5], it can be observed that MCS exhibits higher MI values than VS, and vice versa. Figure 5 depicts that MCS patients exhibit a stronger inter‐channel information interaction when comparing with VS patients. The intensity of inter‐channel information interaction is represented by the darkness of the grid color in the figure. It can be observed that MCS patients exhibit stronger information interaction in the frontal lobe and temporal lobe brain regions in microstate A, in contrast to VS patients. The difference connectivity matrix of microstates B, C, and D generally indicates higher MI values in MCS patients across various brain regions. However, in microstate E, the MI connection value appears stronger in the frontal lobe and parietooccipital brain regions of MCS patients compared to VS patients.

### 3.3 Feature selection and classification results

**FIGURE 6:**
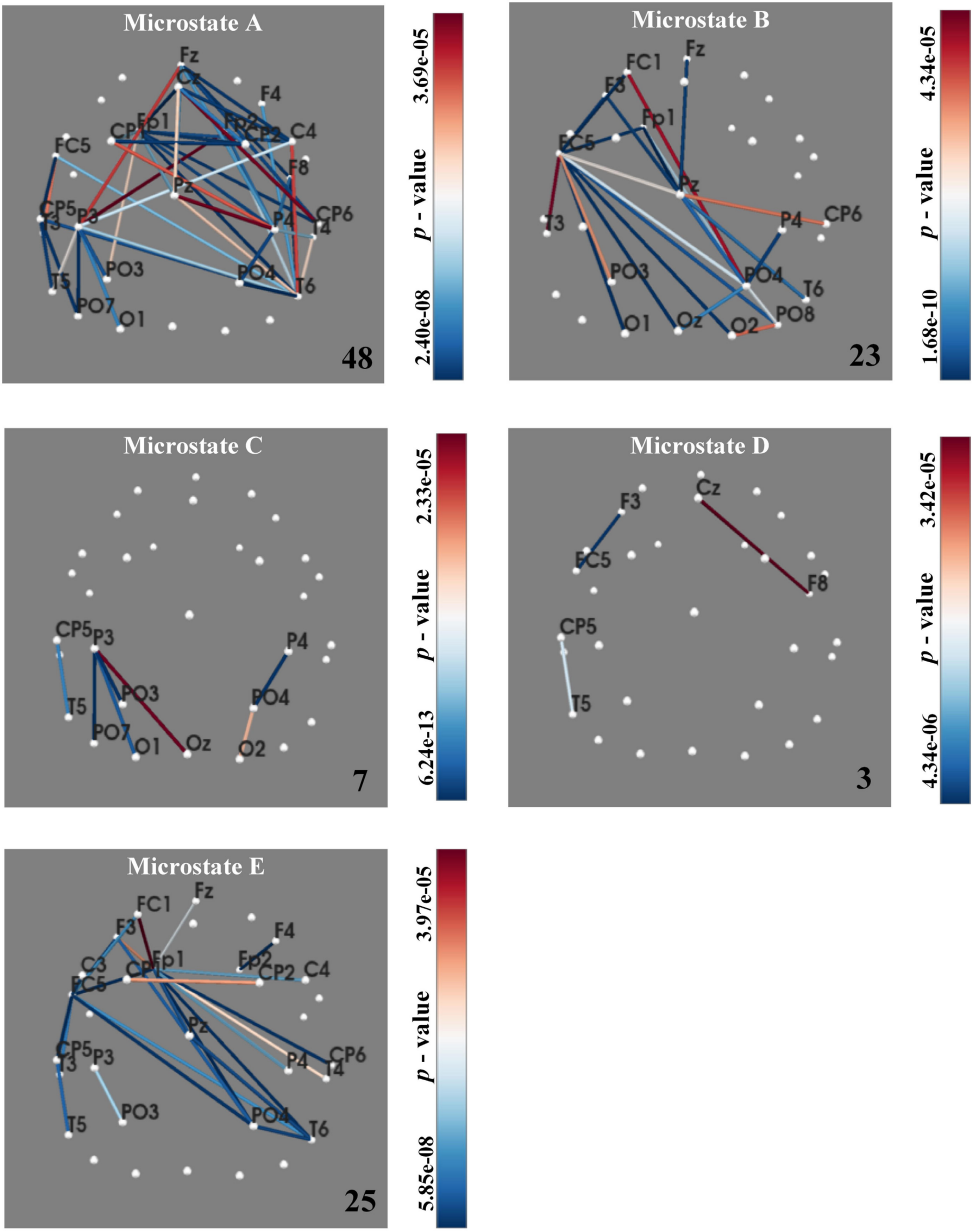
Networks with significant differences, which were selected as the total feature subset. The color bar represents the *p*‐value, gradually increasing from blue to red.

**FIGURE 7:**
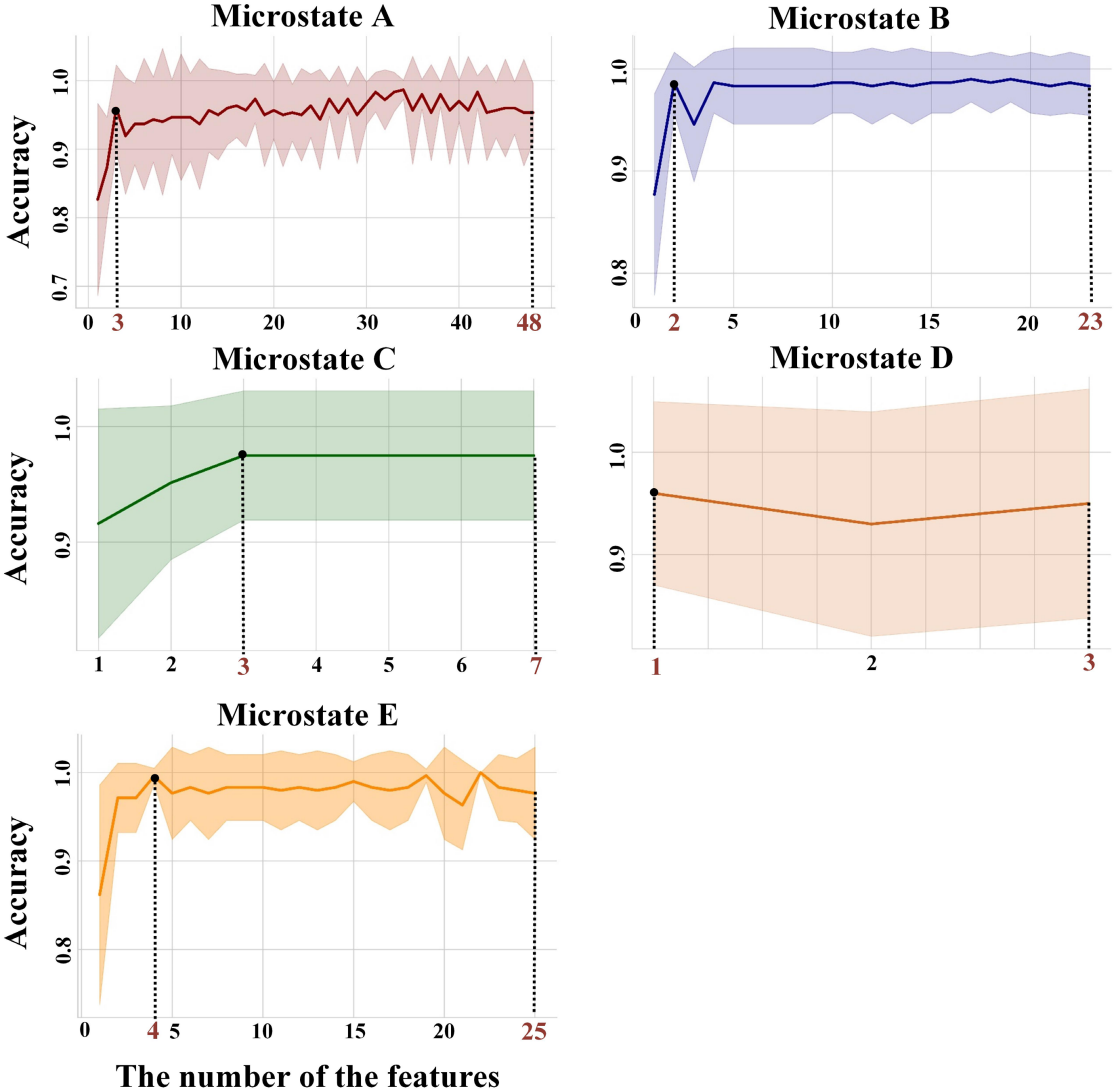
Accuracy for each microstate. The first few dimensional features with the best classification performance were selected as the optimal feature subset. Curve represents the average of the five test accuracies and the ribbon of the curve denotes the SD of using CV.

This study used feature preprocessing to find statistical MI values between two patient groups. Then, TFS was obtained for the five microstates, corresponding to 48‐, 23‐, 7‐, 3‐, and 25‐dimensional statistical difference features, respectively. The networks exhibiting significant differences can be observed in Figure 6. Subsequently, the TFS is processed by the mRMR feature selection algorithm and sent to the CatBoost classifier for hierarchical cross‐validation. The top 3‐, 2‐, 3‐, 1‐, and 4‐dimensional features of each microstate after mRMR feature ranking were selected as the OFS, which were shown in Figure 7. And the classification results on the test set are shown in Table 2. Feature selection allowed us to obtain the OFS, which had fewer dimensions but resulted in the best classification effect. Additionally, the OFS allowed us to prioritize which brain cortex the best features corresponded to, thereby reducing the difficulty of further analysis.

**TABLE 2:** Classification performance (mean ± SD).

MS	Accuracy (%)	Sensitivity (%)	Specificity (%)	OFS|TFS
A	95.05 ± 0.15	91.30 ± 0.17	96.75 ± 0.13	3|48
B	98.12 ± 0.21	94.13 ± 0.18	97.56 ± 0.08	2|23
C	96.49 ± 0.15	94.26 ± 0.16	99.43 ± 0.15	3|7
D	94.55 ± 0.23	97.10 ± 0.11	93.26 ± 0.23	1|3
E	98.35 ± 0.12	97.31 ± 0.13	99.55 ± 0.10	4|25

Abbreviations: MS, microstate; OFS, optimal feature subset; TFS, total feature subset.

### 3.4 MIFCN analysis

**FIGURE 8:**
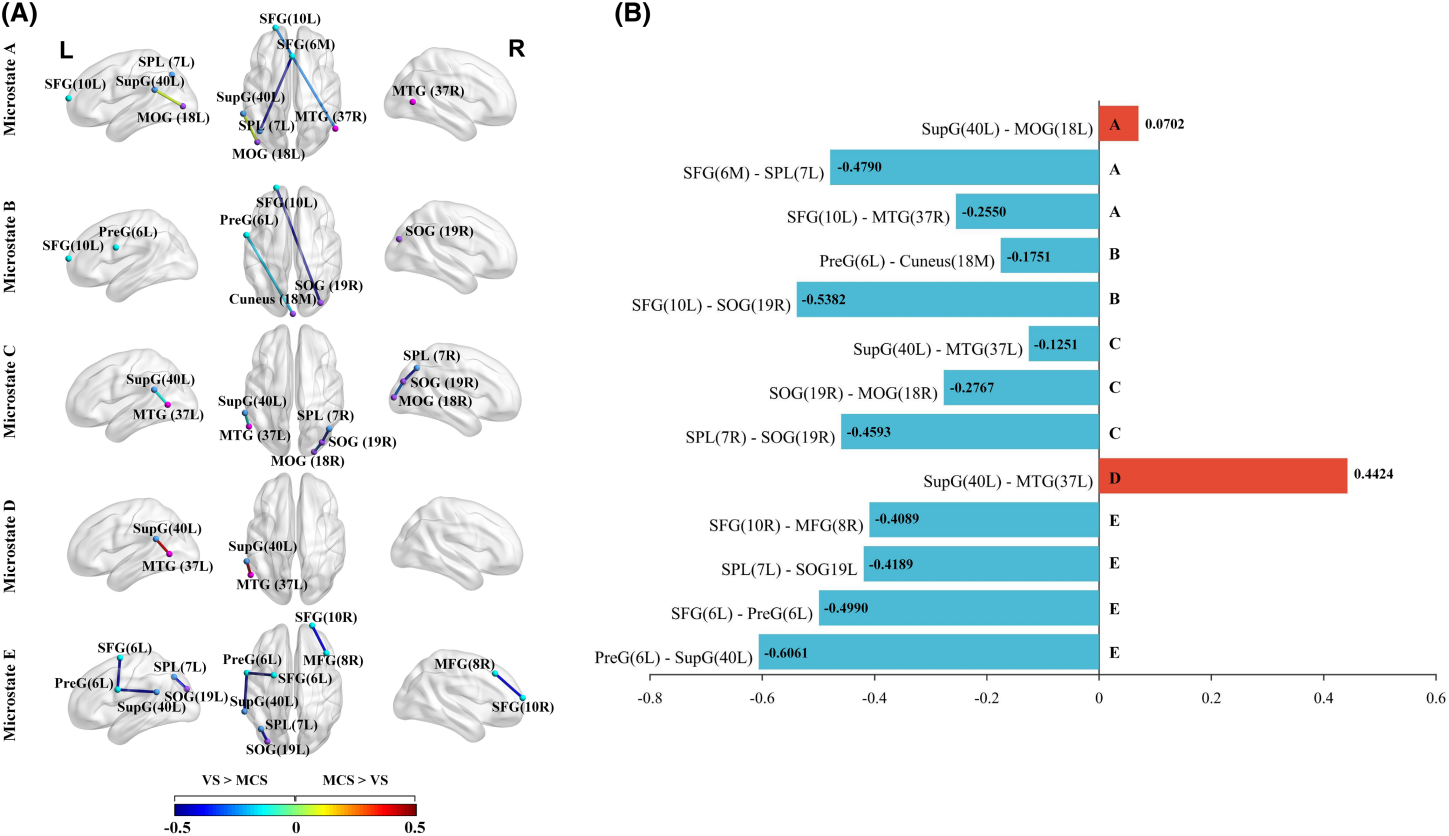
(A) Cortical connections corresponding to optimal feature subset in each microstate. The color of the connecting edges corresponds to the group mean mutual information (MI) difference for each microstate. Cortical connectivity map showing the bilateral sagittal and axial views. Node colors represent different brain regions (frontal lobe in light blue, parietal lobe in dark blue, temporal lobe in pink, and occipital lobe in purple). (B) MI‐based group mean difference values (MCS–VS) in each microstate. The horizontal coordinate indicates the MI value. The vertical axis displays the functional connectivity patterns of various microstates depicted in (A).

Currently, there are two commonly used methods for mapping scalp EEG data to the cerebral cortex, namely automatic mapping methods and source localization methods.^34^, ^35^ The method of automatically mapping EEG signals to the cortex uses the corresponding relationship between the electrode position and the three‐dimensional structure of the cerebral cortex, which is more convenient and intuitive than the source localization method. Therefore, aiming to highlight the different patterns of information flow between the two types of patients in each microstate of the brain more intuitively, this study used the BrainNet Viewer toolkit to draw the cerebral cortex based on the OFS obtained in the previous step. The resulting visualization is shown in Figure 8. In addition, this study also mapped the EEG data to the Brodmann area of the cerebral cortex, as depicted in Table 3. The table provides a detailed description of the microstate corresponding to each electrode and the name of the brain area.

**TABLE 3:** The electrodes in each OFS and their corresponding projection cortical areas.

Labels	Anatomy	Gyri(BA)	Coordinates	Microstates
Frontal cortex				
FP1	Superior Frontal Gyrus	SFG (10L)	−21, 63, 12	A, B
FP2	Superior Frontal Gyrus	SFG (10R)	24, 63, 13	E
FZ	Superior Frontal Gyrus	SFG (6M)	0, 27, 61	A
F4	Middle Frontal Gyrus	MFG (8R)	42, 28, 44	E
FC1	Superior Frontal Gyrus	SFG (6L)	−25, 0, 66	E
FC5	Precentral Gyrus	PreG (6L)	−59, 3, 26	B, E
Parietal cortex				
CP5	Supramarginal Gyrus	SupG (40L)	−62, −46, 23	A, C, D, E
P3	Superior Parietal Lobule	SPL (7L)	−41, −68, 42	A, E
P4	Superior Parietal Lobule	SPL (7R)	44, −66, 43	C
Occipital cortex				
PO3	Superior Occipital Gyrus	SOG (19L)	−33, −84, 27	E
PO4	Superior Occipital Gyrus	SOG (19R)	35, −83, 26	B, C
PO7	Middle Occipital Gyrus	MOG (18L)	−44, −82, 2	A
OZ	Cuneus	Cuneus (18M)	0, −97, 9	B
O2	Middle Occipital Gyrus	MOG (18R)	25, −95, 6	C
Temporal cortex				
T5	Middle Temporal Gyrus	MTG (37L)	−57, −63, 4	C, D
T6	Middle Temporal Gyrus	MTG (37R)	55, −65, 6	A

Abbreviations: BA, Brodmann area; L, left; M, middle; R, right.

As can be seen in Figure 8A, there is an information interaction in the parietooccipital network in microstate A, while microstate B involves more of the frontoparietal network. Microstate C has more activation in the occipital cortex (BA 19R, BA 18) and also involves cortical activation of the parietooccipital and parietotemporal networks. Microstate D involves parietotemporal cortical activation (BA 40L—BA 37L), with close links between parietotemporal regions and dorsal attentional networks. Finally, microstate E activates the frontal cortex more, and there is also information interaction between the frontoparietal and parietooccipital networks. In addition, this study also finds that except for BA 40L—BA 18L in microstate A and BA 40L—BA 37L in microstate D, most of the connections in other microstates indicate that VS patients have stronger information interaction than MCS patients. The MI‐based group‐average difference values can be observed in Figure 8B. The blue column indicates that VS has a stronger MI information flow, and the red column indicates that MCS has a larger MI value.

## 4 DISCUSSION

### 4.1 Spatio‐temporal dynamic analysis

Traditional functional connectivity studies mostly analyzed the neural connections between brain regions based on the principle of stationarity assumption, which obviously ignored the dynamic variability of data. As the field of FC continued to flourish, researchers found that incorporating temporal dynamics into connectivity analysis was critical in elucidating the nature of cognitive processes,^36^, ^37^, ^38^, ^39^ also known as dynamic functional connectivity. The sliding time window method was the most commonly used way to research dFC, but it had numerous limitations in practice because it required specifying the window scale in advance.^40^, ^41^, ^42^ In addition, shorter time windows were incapable of capturing the large‐scale spatiotemporal dynamics of brain networks. Therefore, researchers have been actively seeking methods to analyze the large‐scale network dynamics between brain areas more comprehensively. Microstate is a novel spatiotemporal analysis method that has been applied to electrophysiological data for characterizing brain dynamics and analyzing the dynamic properties of whole‐brain resting‐state EEG data.^10^

This study combined microstate and dFC to study the spatiotemporal state of the brain in DOC patients. To capture the spatiotemporal properties of large‐scale brain networks in DOC patients, a microstate analysis was performed over the entire data length. In order to explore the neural mechanism behind each microstate, the EEG corresponding to each microstate was extracted. Furthermore, to enhance the SNR of the signal used in the calculation of FC, and to investigate the dynamic characteristics of each microstate on a finer scale, the dFC method was applied with a time window length of 30s to lay the foundation for a further data analysis. After calculating the MI in each time window, two data analysis methods were employed. One method involved averaging the MI connectivity matrix in each time window of the five microstates and then performing a subtraction (MCS−VS) to examine the difference in the intensity of information flow in the whole brain between two types of patients. The other method employed statistical analysis to identify connections with significant differences, designated as TFS. TFS was then sent to the classifier for feature selection and classification, in which the first few dimensional features with the greatest contribution served as OFS. We also mapped the cortical connections corresponding to the OFS to the BA area, so that the distribution of these key connections could be seen more intuitively. The results of the two data analysis methods are presented in Figure 2d,e, which correspond to Figures 5 and 8, respectively.

The results of the two kinds of data analysis reflect the brain network information in two dimensions. The connectivity matrix is the data information established on all of the channels, as shown in Figure 5. We can see that the five microstates all reflect a stronger connection of MCS than VS, which is consistent with the previous conventional conclusion.^21^, ^43^ While another data analysis method is based on statistical analysis and feature selection, and its results only focus on a small number of brain network connections in several data dimensions. As depicted in Figure 8, these connections, marked by statistical distinction and high classification accuracy, exhibited a stronger MI flow in VS than MCS patients in microstates A, B, C, and E, except D. These connections seem to be contrary to the previous studies, and we will discuss them further in conjunction with microstates.

### 4.2 Cortical functional connectivity in each microstate of DOC patients

Some studies have pointed out the relations between microstates and corresponding networks, in which microstate A relating to auditory network, microstate B to visual network, microstate C to visual network, microstate D to attention network, and microstate E to default network.^10^, ^44^ Following statistical analysis and feature selection, OFS generally revealed that VS patients had stronger connections. Additionally, these connections of OFS not only had a specific link with the brain‐network involved in the microstate itself but also could well separate the two types of patients. It might also indicate an underestimated level of consciousness in VS patients.

Microstate A, closely related to auditory networks and the temporal lobe,^10^, ^44^ has been further elucidated through studies on auditory consciousness, involving the frontotemporal network.^45^, ^46^ Schmithorst et al.^47^ revealed that children with profound unilateral hearing loss had exhibited smaller activation in regions such as the middle temporal gyrus (BA 37), demonstrating differences in monaural hearing in cross‐modal modulation of visual processing pathways. Brancucci et al.^45^ had also observed hemodynamic activity accompanying auditory conscious experience in regions such as superior frontal gyrus (BA 10) and medial frontal gyrus (BA 6), and had shown that SFG—MFG and insula were important cortical nodes for auditory conscious experience. The FC between BA 10L—BA 37R in this study indicated that the frontotemporal network related to auditory consciousness could be used as an informative feature to distinguish patients with two types of DOC. Additionally, the parietal cortex had contributed significantly to decoding differences in consciousness.^48^ And for patients with DOC, EEG features associated with the frontoparietal network consistently showed a high correlation with consciousness, playing an important role in altered states of consciousness.^49^ Based on the auditory tasks studying working memory, it was pointed out that the left superior parietal lobule (BA 7L) was selectively activated.^50^ The bilateral inferior parietal cortex (BA 40) was preferentially activated for attention to semantic and phonological relationships.^51^ The memory‐related primary visual cortex (containing middle occipital gyrus, BA 18) could respond to unconscious processing.^52^ In this study, BA 6—BA 7L showed a stronger connection in VS patients, indicating that the frontoparietal network of VS patients was activated. The connection between BA 40L and BA 18L exhibited a larger value in MCS patients, which may have indicated that the parietooccipital network in MCS patients had a stronger performance in processing semantic information and visual information.

In Microstate B, associating with the bilateral inferior occipital gyrus and visual networks,^10^, ^44^ our study found that connections involving the frontal and occipital lobes were more pronounced in VS patients, indicating an activated frontooccipital network. These findings are consistent with previous observations of behavioral signs of visual awareness in VS patients.^53^, ^54^ In addition, according to the regulatory function, the brain network could be divided into high‐order networks (Salience network, SN, Dorsal attention network, DAN, Default mode network, DMN) and low‐order networks related to perceptual processing (auditory network, AN, visual network, VN).^55^ Primary visual cortex (BA 18,19) was significantly activated in tracking visual motion patterns,^52^ gestural finger differentiation, sustained attention to color or shape, and horizontal sweep eye movements. The BA 6L–BA 18M and BA 10L–BA 19R connections may have reflected that recursive processing from higher‐order cortex to lower‐order visual cortex in microstate B associated with the visual network plays a key role in distinguishing the two groups of patients.

Microstate C was widely pointed out to be closely related to the saliency network (SN),^10^, ^44^ which was involved in conflict detection and information integration, and contributed to conscious perception.^20^, ^56^ However, the FC of the SN was reduced in MCS patients and barely identifiable in VS patients.^20^ In this study, the corresponding brain network connections in the two types of patients in microstate C involved more occipital cortex, and there was also information interaction between the parietooccipital network and the parietotemporal network. BA 7R was shown to be associated with visuospatial processing and triggered visual attention mechanisms,^57^ and it was also associated with memory,^58^ prompting the brain to recall consciously. BA 7R–BA 19R showed strong connectivity in VS patients, which may have indicated that VS patients were regaining consciousness through visual modulation. From the analysis in microstate B, we know that BA 18 and BA 19 are involved in the primary visual cortex. These results also indicate that the connection in microstate C may be related to the regulation of the visual network in VS patients. In addition, both BA 40L and BA 37L were involved in language and memory.^59^ BA 40L was also related to execution control and conflict detection,^60^ which was similar to the function corresponding to the saliency network corresponding to the microstate C. What's more, BA 37L was associated with deductive reasoning,^61^ and BA 40L—BA 37L may have indicated that the salience network in VS patients under microstate C was also activated.

Microstate D was previously linked to the attention network.^10^, ^44^ The dorsal attentional network (DAN) was an important component of the “task‐positive” network, which was distributed on both sides of the brain. It was associated with the ventral frontal cortex and temporoparietal regions, including the temporoparietal junction (TPJ).^62^ Posner et al.^63^ studied the effect of parietal lobe damage on attention and found that parietal lobe damage could lead to the phenomenon of separation of attention. There were also several studies showing that DAN was activated in attention shifting, eye movement and hand–eye coordination.^64^ This study revealed a connection between BA 40L and BA 37L in microstate D, which was the connection between the parietal and temporal lobes. In addition to being related to language and memory, BA 40L could also participate in target processing,^65^ and BA 37 could participate in deductive reasoning. These tasks required the participation of the attention mechanism. Different from this, a brain network connection was also contained in microstate C. In this study, the stronger connections on MCS patients may have indicated that the attention network of MCS patients in microstate D was correspondingly activated.

Microstate E mainly consisted of three main subregions: medial forehead, posterior cingulate gyrus, and inferior parietal lobule.^33^ The medial forehead was associated with self‐evaluation; and the posterior cingulate gyrus was associated with self‐awareness. The inferior parietal lobule was associated with memory. This left–right symmetric microstate had also been shown to be related to the default network (DMN) in some microstate studies.^27^, ^33^ DMN activity was closely related to the patient's level of consciousness and was considered as an important indicator to distinguish MCS from VS.^66^, ^67^ Numerous previous studies had shown that default network connectivity was impaired in DOC, with more retention in MCS than in VS. Boly et al.^22^ found that the default network of VS still had residual FC whereas the default network was not present in brain‐dead patients. Vanhaudenhuyse et al.^21^ pointed out that the connection strength of the default network was correlated with the level of consciousness: the lower the consciousness, the weaker the connection. In this study, microstate E was mainly involved in connections between the frontal lobes, and between the frontoparietal and parietooccipital networks. The functions involved in BA 6L were very diverse, such as object naming, episodic long‐term memory, and visuospatial attention.^68^ The key connections involved in microstate E included BA 6L–BA 6L and BA 6L–BA 40L, which may have indicated that VS patients were also activated correspondingly in the frontal cortex. BA7L could be involved in temporal context recognition, and BA 19L had also been shown to reason based on visual and semantic attributes. BA 10R could trigger prospective memory based on events and time, and could respond to painful thermal stimuli.^69^ BA 8R could participate in memory retrieval and could respond to related stimuli.^70^ Taken together, BA 7L–BA 19L and BA 10R–BA 8R may have indicated that VS patients were not unconscious when faced with pain or stimuli, but could activate relevant brain regions and respond to stimuli.

## 5 CONCLUSION

This study represented a pioneering exploration into the temporal and spatial variability of dynamic microstate brain networks in DOC. Our results demonstrated the effectiveness of utilizing microstate‐based features to differentiate between two distinct DOC patient categories. The stronger activation of specific brain regions in different microstates in VS patients suggested that their level of consciousness may have been underestimated. These findings may contribute to the understanding of the neurophysiological mechanisms underlying human consciousness and provide new insights into revealing the level of consciousness in DOC.

## FUNDING INFORMATION

This work was supported by the National Natural Science Foundation of China (No. 61773408 and No. 82272118) and the Beijing Natural Science Foundation (No. 23G10455).

## CONFLICT OF INTEREST STATEMENT

The authors declare that they have no known competing financial interests or personal relationships that could have appeared to influence the work reported in this paper.

## Supporting information


Data S1.

## Data Availability

The data that support the findings of this study are available on request from the corresponding author. The data are not publicly available due to privacy or ethical restrictions.
